# Mechanistic rationales for combining immunotherapy with radiotherapy

**DOI:** 10.3389/fimmu.2023.1125905

**Published:** 2023-06-12

**Authors:** Alexander Chi, Nam Phong Nguyen

**Affiliations:** ^1^ Department of Radiation Oncology, Capital Medical University Xuanwu Hospital, Beijing, China; ^2^ School of Basic Medical Sciences, Capital Medical University, Beijing, China; ^3^ Department of Radiation Oncology, Howard University, Washington, DC, United States

**Keywords:** radiotherapy, immunotherapy, immune checkpoint inhibitors, tumor immune micro-environment, cancer

## Abstract

Immunotherapy consisted mainly of immune checkpoint inhibitors (ICIs) has led to significantly improved antitumor response. However, such response has been observed only in tumors possessing an overall responsive tumor immune micro-environment (TIME), in which the presence of functional tumor-infiltrating lymphocytes (TILs) is critical. Various mechanisms of immune escape from immunosurveillance exist, leading to different TIME phenotypes in correlation with primary or acquired resistance to ICIs. Radiotherapy has been shown to induce antitumor immunity not only in the irradiated primary tumor, but also at unirradiated distant sites of metastases. Such antitumor immunity is mainly elicited by radiation’s stimulatory effects on antigenicity and adjuvanticity. Furthermore, it may be significantly augmented when irradiation is combined with immunotherapy, such as ICIs. Therefore, radiotherapy represents one potential therapeutic strategy to restore anti-tumor immunity in tumors presenting with an unresponsive TIME. In this review, the generation of anti-tumor immunity, its impairment, radiation’s immunogenic properties, and the antitumor effects of combining radiation with immunotherapy will be comprehensively discussed.

## Introduction

Although immunotherapy for cancer has been intensely studied, significant and durable antitumor response with limited severe treatment-related toxicity has only been observed since FDA’s approval of immune checkpoint inhibitors (ICIs) that target specific immune checkpoints (1–3). Over the past decade, ICIs have quickly become the primary systemic treatment option for advanced melanoma and non-small cell lung cancer (NSCLC) with expanding indications for solid tumors arising from other anatomical sites (2–5). ICIs that are currently in clinical use mostly target the PD-1/PD-L1(PD-(L)1) immune checkpoint at the site of peripheral tumor (6). This strategy of targeting the primary mechanism of escape from cancer immunosurveillance at the peripheral tumor sites has led to the restoration of adaptive anti-tumor immunity in an overall immunosuppressive local tumor micro-environment (TME) without causing a significant systemic response in normal tissues (1, 7). Thus, leading to long lasting anti-tumor response and a low incidence of severe toxicities in a number of patients (4, 5). Based on previous studies on the treatment of solid tumors with anti-PD-(L)1 inhibitors, the tumor immune micro-environment (TIME) of responders to ICIs has been characterized in contrast to non-responders (8–13). An overall inflamed TIME in which IFN-γ driven tumor expression of PD-L1, and tumor infiltration by functional CD8^+^ T cells have been consistently identified in responders (8, 12, 13). On the contrary, a paucity of tumor infiltration by functional CD8^+^ T cells has been clearly demonstrated in non-responders. Tumor progression after an initial response may also occur after acquiring additional mechanisms of immune evasion, such as loss-of-function mutations or other genomic alterations, which can subsequently induce an overall non-inflamed TIME (14–17). Currently, the response rate to ICI remains low in non-lymphoid solid tumors, such as NSCLC (5, 17). This makes more effective treatment strategies to overcome ICI resistance urgently needed. With increased understanding of the mechanisms of antitumor immunity and its impairment, many strategies to enhance antitumor immunity and overcome immunosuppression within the TIME, subsequently converting a non-inflamed or “cold” TIME into an inflamed one have been proposed (18–20). As one major local treatment strategy for cancer, radiotherapy (RT) has been shown to have immunostimulatory properties, which can be further exploited to serve this purpose (21–25). In this review, the impairments leading to suboptimal antitumor immunity and poor response to ICIs, RT’s immunogenic properties, and the rationales for combining RT with immunomodulatory agents to remodel the TIME in order to restore or augment anti-tumor immunity will be discussed.

## The underlying mechanism of antitumor immunity leading to an inflamed TIME 

**Figure 1:**
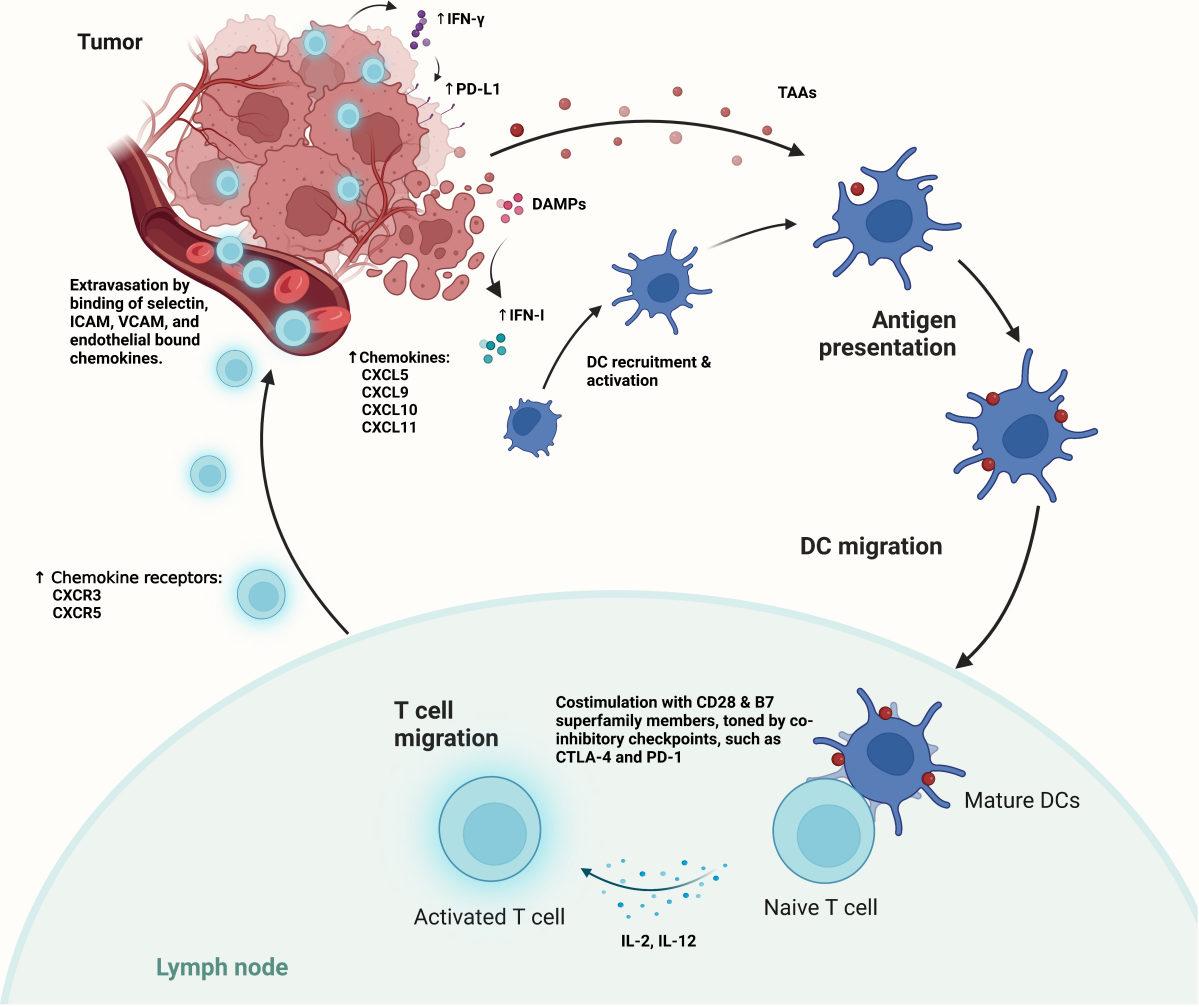
The generation of antitumor immunity relies on the detection of tumor antigens and danger-associated molecular patterns (DAMPs) by antigen presenting cells, such as dendritic cells (DCs). The presence of DAMPs leads to increased IFN-I secretion in the TIME, and the subsequent recruitment and activation of DCs. Activated DCs migrate to the tumor draining lymph nodes (tdLNs), where they cross present tumor antigens, and express additional co-stimulatory checkpoints and cytokines for T cell activation. Such activation may be attenuated by the presence of co-inhibitory checkpoints, such as CTLA-4 and PD-1. Upon activation, tumor-antigen specific T cells with increased expression of chemokine receptors migrate to the peripheral tumor, which have increased expression of T cell specific chemokines. Effector T cells then extravasate from the tumor vasculature through the binding of selectin, as well as the binding and activation of integrins, such as ICAM and VCAM for adhesion and transmigration into the tumor micro-environment.

Immune surveillance of cancer has long been known to exist, which allows the immune system to eradicate malignant lesions as they arise in the human body (7, 26, 27). To initiate anti-tumor immunity for tumor elimination (**Figure 1**), a coordinated activation of both innate and adaptive immunity is required (26–29). In the process, tumor antigens and the detection of “danger signals” from the tumor by the innate immune sensors, trigger the recruitment and activation of antigen presentation cells (APCs) (28–33). Subsequently, tumor antigen cross-presentation by APCs that travel to the tumor draining lymph node (tdLN) leads to the priming and activation of T cells (33). Co-stimulation signals, which may be further tuned by co-inhibitory signals, are required for T cells to be fully activated; while sub-optimal activation only leads to T cell anergy (34, 35). Fully activated T cells are able to express a unique set of “homing” chemokine receptors that are accompanied by increased expression of related chemokines in the TME. Thus, allowing for the homing of activated T cells to the tumor and its infiltration (33). One well known example is the CXC receptor 3(CXCR3), and its ligands CXCL9, CXCL10, and CXCL11, which interactions in tumor infiltration may also involve other chemokines, such as CXCL5 (33, 36, 37). At the site of tumor, selectin ligands and other adhesion molecules are also required for the binding of blood vessels and extravasation by T cells (33, 36). After tumor infiltration, cytotoxic CD8^+^ T cells will recognize tumor cells through recognizing tumor antigens presented by the major histo-compatibility complex class I (MHC-I) molecules and induce T cell mediated cytotoxicity (33).

Immune escape by tumor cells eventually develops. T cell activation within the TIME will stimulate the expression of PD-1 and release of IFN-γ by T cells (2). IFN-γ then induces PD-L1 expression by tumor cells as well as other cells within the TIME, resulting in the inhibition of the local immune response through PD-1/PD-L1 interaction (2, 38, 39). The process of PD-1/PD-L1 mediated effector T cell exhaustion is also called adaptive immune resistance. It serves as a dominant feedback mechanism to maintain peripheral tolerance, and is a common mechanism of immune escape by various cancers (2, 6, 40). Also, it makes re-invigoration of tumor infiltrating cytotoxic T lymphocytes (CTLs) within the TIME through targeting the PD-1 axis a very effective therapeutic strategy for cancer (1–6, 8, 41). An inflamed TIME that is characterized by IFN-γ induced PD-L1 expression by mostly tumor cells, and the presence of functional tumor infiltrating lymphocytes (TILs) is often associated with such re-invigoration, which is only observed in a limited number of patients with cancers such as NSCLC (2, 8, 41). This implies the presence of other impairment(s) within the steps of anti-tumor immunity generation.

## Major impairments of anti-tumor immunity leading to a noninflamed TIME

**Figure 2:**
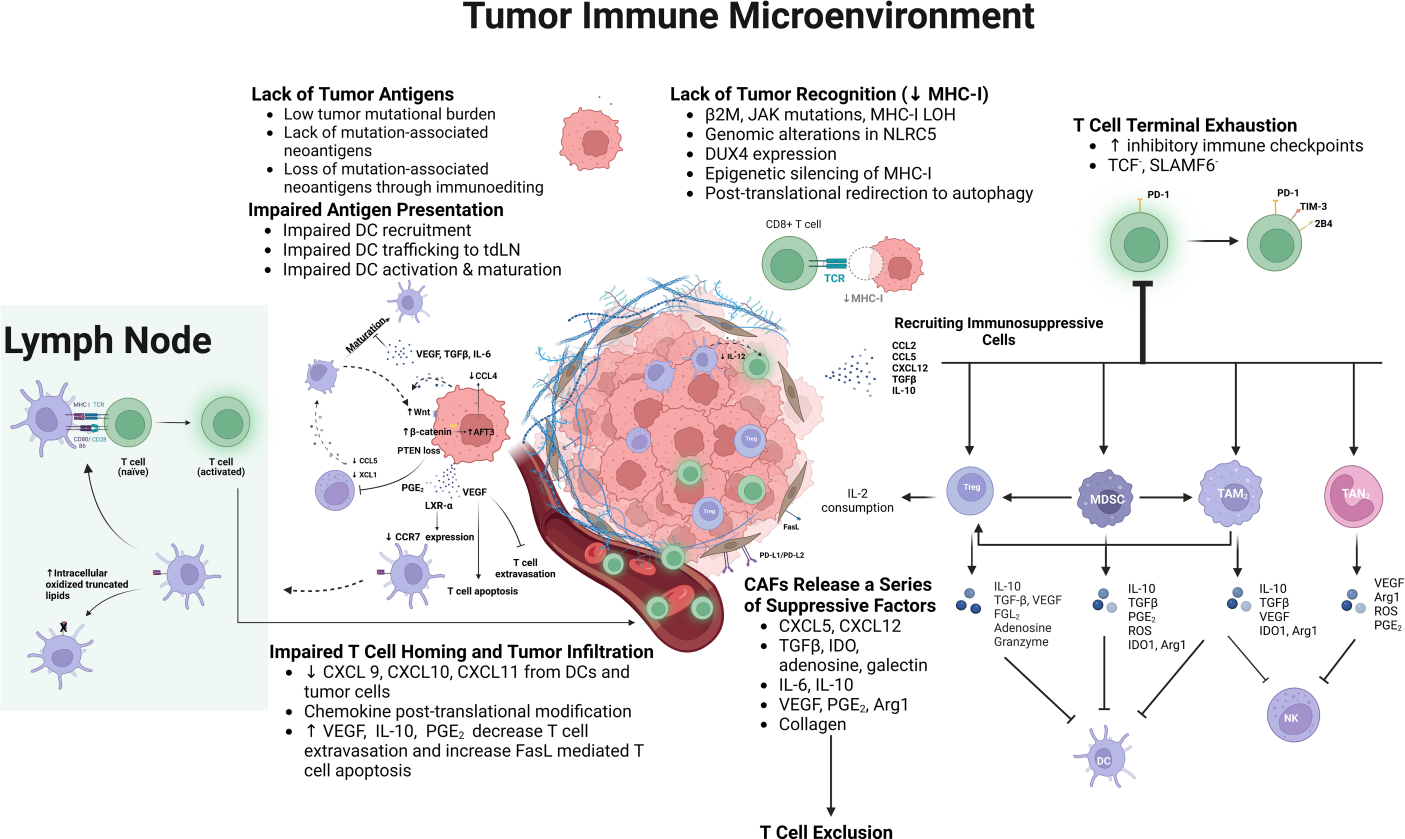
Underlying mechanisms of impaired antitumor immunity generation include: lack of tumor antigens, which develops overtime due to immunoediting; impaired antigen presentation resulting from impaired dendritic cell (DC) trafficking and maturation due to various tumor intrinsic mechanisms, including PTEN loss, Wnt/β-catenin expression, increased expression of LXR-α ligands, as well as increased prostaglandin E2 (PGE_2_), vascular endothelial growth factor (VEGF), IL-6, and TGFβ expression; suboptimal T cell co-stimulation; impaired cytotoxic T lymphocyte (CTL) homing and tumor infiltration due to decreased chemotactic chemokines in the tumor immune micro-environment (TIME), and down-regulation of adhesion molecules and increased Fas ligand expression in the tumor vasculature; exclusion and inhibition of CTLs by stromal cells, such as cancer associated fibroblasts (CAFs); lack of MHC-I expression by tumor cells resulting from genomic alterations of MHC-I or any component of the antigen presenting machinery, NOD-like receptor family, caspase recruitment domain containing 5 (NLCR5), embryonic transcription factor, DUX4, epigenetic silencing, and post-translational re-direction to autophagy; terminal exhaustion of CD8^+^ T cells within the TIME characterized by the lack of TCF and SLAMF6 expression, increased expression of PD-1, and additional inhibitory checkpoints, such as TIM-3 and 2B4; and the recruitment of suppressive immune cells, such as Tregs, myeloid derived suppressor cells (MDSCs), as well as macrophages and neutrophils with their polarization toward the M_2_/ N_2_ phenotype. These suppressive cells inhibit CTLs, DCs, and NK cells, while the suppressive mediators they release into the TIME further stimulate their recruitment and expansion within the TIME.

Various mechanisms of immune escape by cancer leading to a paucity of T cell infiltration or a lack of activated T cells within the TME have been characterized in recent years (17, 33, 42–44). Major impairments in the generation of anti-tumor immunity, include the paucity of tumor neoantigens, impaired antigen presentation, lack of T cell priming and activation, poor tumor infiltration by activated T cells, poor tumor cell recognition/impaired IFN-γ signaling, and alterations in the composition and properties of immune cells within the peripheral TIME (**Figure 2**).

Mutations arise and accumulate during carcinogenesis and cancer progression, leading to the generation of tumor neoantigens (45, 46). Such neoantigens induce T cell specific reactivity, especially in patients who respond to immune checkpoint blockade (47–49). A high tumor mutation burden (TMB) consistently correlated with a higher incidence of durable clinical response (DCR) and improved survival after treatment with anti-CTLA-4 or anti-PD-L1 ICIs for cancers which are known to harbor a higher level of somatic mutations (50–54). This observation is due to the higher likelihood of a high neoantigen load in the presence of a high TMB (55). As shown in a cohort of melanoma patients, DCR to ICIs is not guaranteed by the presence of a high TMB (50). Instead, specific mutation associated neo-epitopes are required to elicit a cytotoxic anti-tumor T cell response in long-term responders to ICIs, and their occurrence is low even in the presence of a high TMB. This is due to immunoediting or the loss of tumor clones which harbored these mutant neoantigens over time (50, 52, 55). Subsequently, immune ignorance develops with poor TIL infiltration of the tumor. As shown in NSCLC, the repression of clonal neoantigen expression is ongoing in an inflamed TIME through various mechanisms, such as DNA copy number loss, suppression of gene transcription, epigenetic, as well as post-translational modification (56). Loss of immunogenic tumor mutation associated neoantigens has also been identified as one mechanism of acquired resistance to ICIs in NSCLC patients who underwent treatment with anti-PD-1 or combined anti-PD-1 and anti-CTLA-4 ICIs (57). Additional strategies to induce or maintain an adequate immunogenic mutant neoantigen repertoire in cancer patients are needed. However, high TMB and increased PD-L1 expression together represent key characteristics of an immunogenic tumor that has developed PD-1 axis mediated adaptive immune resistance, which may be used to select patients for treatment with anti-PD-1 or anti-CTLA-4 ICIs (50, 52).

As evidenced by a lack of DCR to ICIs in the presence of very high TMB, additional mechanisms of immune escape leading to a paucity of TILs within the TIME exist (50, 52). The antigen cross-presentation process, a key step in the generation of adaptive anti-tumor immunity, can be impaired in multiple ways, leading to a non-inflamed TIME. Among them, the repression of type I conventional dendritic cell (cDC_1_) trafficking into the TME due to tumor-intrinsic activation of the WNT/β-catenin signaling pathway has been well studied (58–61). A paucity of CD103^+^ DCs was observed when WNT/β-catenin was expressed in melanoma, which was due to the higher expression of transcriptional repressor AFT3. As a result, AFT3 inhibited the expression of CCL4, which is the key chemokine for guiding cDC_1_ migration into the tumor (58). Activated b-catenin signaling due to mutations or somatic copy number alteration is frequently observed in non-inflamed tumors (59). As shown in NSCLC, b-catenin expression is associated with shorter overall survival (OS) and significantly less tumor infiltrating CD8^+^ T cells, especially in the presence of a high TMB (60, 61). The trafficking of DCs into the tdLN can also be suppressed by tumor intrinsic alterations, such as increased expression of liver X receptor-α (LXR-α) ligands that would bind to LXR-α on DCs to inhibit CCR7 expression, which is required for DC migration to the tdLN (62, 63).

Other than the disruption of DC trafficking, activation and maturation of DCs can also be impaired. The maturation of DCs is induced through the detection of danger associated molecular patterns (DAMPs), which are mostly composed of intracellular proteins, and pieces of cytosolic tumor DNA from dying tumor cells by innate immune sensors (29, 64, 65). This leads to increased IFN-β signaling, which induces downstream stimulation of antigen presentation, DC maturation and trafficking to the tdLN. The innate immunity induced by DAMP or cytosolic DNA may be attenuated in tumors with increased cyclooxygenase-driven prostaglandin E2 (PGE_2_) production, leading to decreased DC recruitment and maturation (66, 67). On the other hand, decreased innate immunity may also result from the neutralization or inhibition of DAMP signaling, which inhibits subsequent DC activation (68, 69).

DC recruitment into the tumor, and differentiation are also mediated through NK cells, which produce cDC_1_ chemo-attractants CCL5, CXCL1, and survival stimulating cytokine FLT3LG. However, NK cells’ viability and chemokine production may be suppressed by tumor PGE_2_ overexpression, which demonstrates how the tumor may exert its influence on the TIME (70, 71). Other intrinsic tumor factors may also suppress DC maturation, such as vascular endothelial growth factor (VEGF); or induce a tolerogenic DC phenotype (DCreg), such as interleukin (IL)-6, transforming growth factor (TGF)β (72). At last, cross presentation by DCs can also be impaired due to the accumulation of oxidized lipids as a result of oxidatively truncated lipids’ binding the heat shock protein (hsp) 70, which prevents the translocation of peptide-MHC complex to the cell surface (73, 74). Activation of CD8^+^ T cells also requires additional stimulatory signals from the APCs, including co-stimulatory signals and APC-released cytokines (64, 75). Peripheral tolerance can develop with suboptimal co-stimulation, which may be alleviated by agonist antibodies to the co-stimulation checkpoints, activating cytokines, or inhibitors to inhibitory checkpoints, such as CTLA-4 (33).

Chemo-taxis of effector T cells to the tumor depends on the expression of CXCR3 on T cells and the presence of its ligands, CXCL9, CXCL10, and CXCL11 within the TIME (76). Within the tumor, Batf3-dependent cDC_1_s that express CXCL9 and CXCL10 are required for effector T cell chemo-taxis (77). A lack of tumor infiltration by T cells is observed in their absence. On the contrary, their presence is associated with a state of T cell exhaustion, and response to ICIs (78). More recently, macrophage-derived CXCL9 and CXCL10 were also found to be required for CD8^+^ T cell infiltration into the TIME, and any response to ICIs (79). Chemokines can be modified by post-translational modifications, which have been associated with impaired T cell chemo-taxis to the tumor (33, 80).

In addition, T cell extravasation and tumor infiltration can be suppressed by endothelial cells and stromal cells. CTLs must adhere to the endothelial lining for extravasation, and this may be inhibited by the vascular endothelial growth factor (VEGF) mediated down-regulation of adhesion molecules, or the induction of CTL apoptosis through Fas ligand (FasL) overexpression that are induced by VEGF, IL-10, or PGE_2_ within the TME (81, 82).

The tumor stroma has largely been known to be immunosuppressive, which can form dense areas of fibrosis in advanced cancer, leading to a state of poor T cell infiltration into the tumor (83). Increased collagen density has been associated with decreased CD8^+^ T cell infiltration into the tumor (84). Similarly, cancer associated fibroblasts (CAFs) have been shown to induce an immunologically excluded phenotype mediated by increased TGFβ signaling within fibroblasts (85). Other mechanisms of CD8^+^ T cell inhibition by CAFs include CXCL12 mediated CTL exclusion; PD-L1/L2, FasL upregulation; generation of adenosine; IL-6 mediated DC conversion to DCreg; and blunting of TCR signal transduction (86). In addition, CAFs can secret immunosuppressive factors and stimulate the recruitment of suppressive immune cells into the TIME (87). Tumor stroma along with the ill-formed tumor vasculature represent areas of great therapeutic potential for the generation of a more immunogenic TIME (83, 87, 88).

Reduced tumor neoantigen recognition may result from decreased MHC-I expression caused by the presence of genomic alterations of MHC-I or any component of the antigen processing machinery (APM), such as β2-microglobulin (B2M) (72, 89). Alternatively, it may result from the reduced MHC-I up-regulation due to impaired IFN-γ signaling caused by JAK1/2 mutations (14). Complete loss of the B2M gene causes complete loss of MHC-I expression (14, 15). Genomic alterations of B2M and JAK1/2 have been associated with acquired resistance to ICIs along with MHC-I loss of homozygosity (LOH) (14, 15, 90, 91). Other mechanisms leading to decreased MHC-I expression also exist, which include genomic alterations of the transactivator of MHC-I related genes, NLCR5 (NOD-like receptor family, caspase recruitment domain containing 5); the expression of embryonic transcription factor, DUX4; epigenetic silencing; and post-translational re-direction to autophagy (72, 92, 93).

At last, alterations of the immune cell composition within the TIME can lead to suppression of anti-tumor immunity. One such alteration is the increased presence of terminally exhausted CD8^+^ T cells. Initially observed after chronic antigen exposure, T cell exhaustion is characterized by increased PD-1 expression its co-expression with additional inhibitory checkpoint receptors, and hierarchical loss of cytolytic function in CD8^+^ T cells (94–97). TILs appear to be at different stages of exhaustion with effector functions preserved in progenitor exhausted CD8^+^ T cells, but lost in terminally exhausted CD8^+^ T cells (95–97). Progenitor exhausted T cells appear to have greater chromatin accessibility; higher expression of stimulatory cytokines, co-stimulatory checkpoints, and survival/memory molecules; while terminally exhausted T cells had more accessibility to and expression of co-inhibitory receptors, effector molecules, and transcription factors associated with exhaustion (98). As shown in NSCLC, increased degree of CD8^+^ T cell exhaustion is correlated with increased PD-1 expression and the co-expression of additional co-inhibitory receptors, such as TIM-3, TIGIT, and CTLA-4 at later stages of cancer progression (99, 100). PD-1 appears to be the primary mediator of CD8^+^ T cell exhaustion. However, anti-tumor response to anti-PD-1 therapy alone is poor in tumors infiltrated by terminally exhausted TIL’s, which mostly reside in the TIME (97–100). On the contrary, combined blockade to PD-1 and other co-inhibitory checkpoints was shown to restore effector function in terminally exhausted TILs, and generate significant anti-tumor activity when PD-1 and additional co-inhibitory checkpoints are co-expressed (97, 100).

The immune cell composition may also be sculpted to have an increased presence of suppressive immune cells within the TIME. The inhibitory roles of regulatory T cells (Tregs), and myeloid derived suppressor cells (MDSCs) have been well characterized, while tumor associated macrophages (TAMs) and tumor associated neutrophils (TANs) can develop pro-tumor or anti-tumor properties (101–106). Tregs, Forkhead box P3 (FOXP3) expressing CD4^+^ T cells, are recruited into the TIME through up-regulation of CCR4, CCR5, CCR8, and CCR10 mediated chemotaxis in inflamed tumors by both tumor and immune cells; and CCR4 mediated chemotaxis due to increased CCL22 expression in EGFR mutant non-inflamed tumors, exert their inhibitory effects on CTLs through IL-2 depletion, binding and capturing of co-stimulatory signals on dendritic cells, and the production of immunosuppressive factors, such as TGFβ, IL-10, FGL_2_, VEGF, and granzymes (101, 102). MDSCs are derived from polymorphonuclear (PMN) or monocytic myeloid cells in the presence of cancer induced inflammation (103, 104). They secrete a series of immunosuppressive mediators, such as IL-10, PGE_2_, TGFβ, free radicals; inducing M_2_ macrophages and Tregs, while suppressing CTL adhesion, TCR expression, activation, and survival (105, 106). TAM and TAN may polarize into immunostimulatory/anti-tumor, or immunosuppressive/pro-tumor types by stimulatory signals, such as IFN-γ, or suppressive signals, such as, TGFβ (105–107). While type 1(M_1_) macrophages have potent antigen presentation and phagocytotic properties, type 2 (M_2_) macrophages are immunosuppressive for which they express inhibitory immune checkpoints, such as PD-L1; and secrete a series of suppressive factors, such as TGFβ, IL-10, IDO, and arginase 1 (Arg1), which stimulate Treg function, inhibit DC maturation, and suppress CTL function (105, 106). Similar to TAMs, TANs can secrete a series of immunosuppressive mediators and express a series of inhibitory immune checkpoints, once induced by suppressors, such as TGFβ (107).

## The immunomodulatory role of radiotherapy

**Figure 3:**
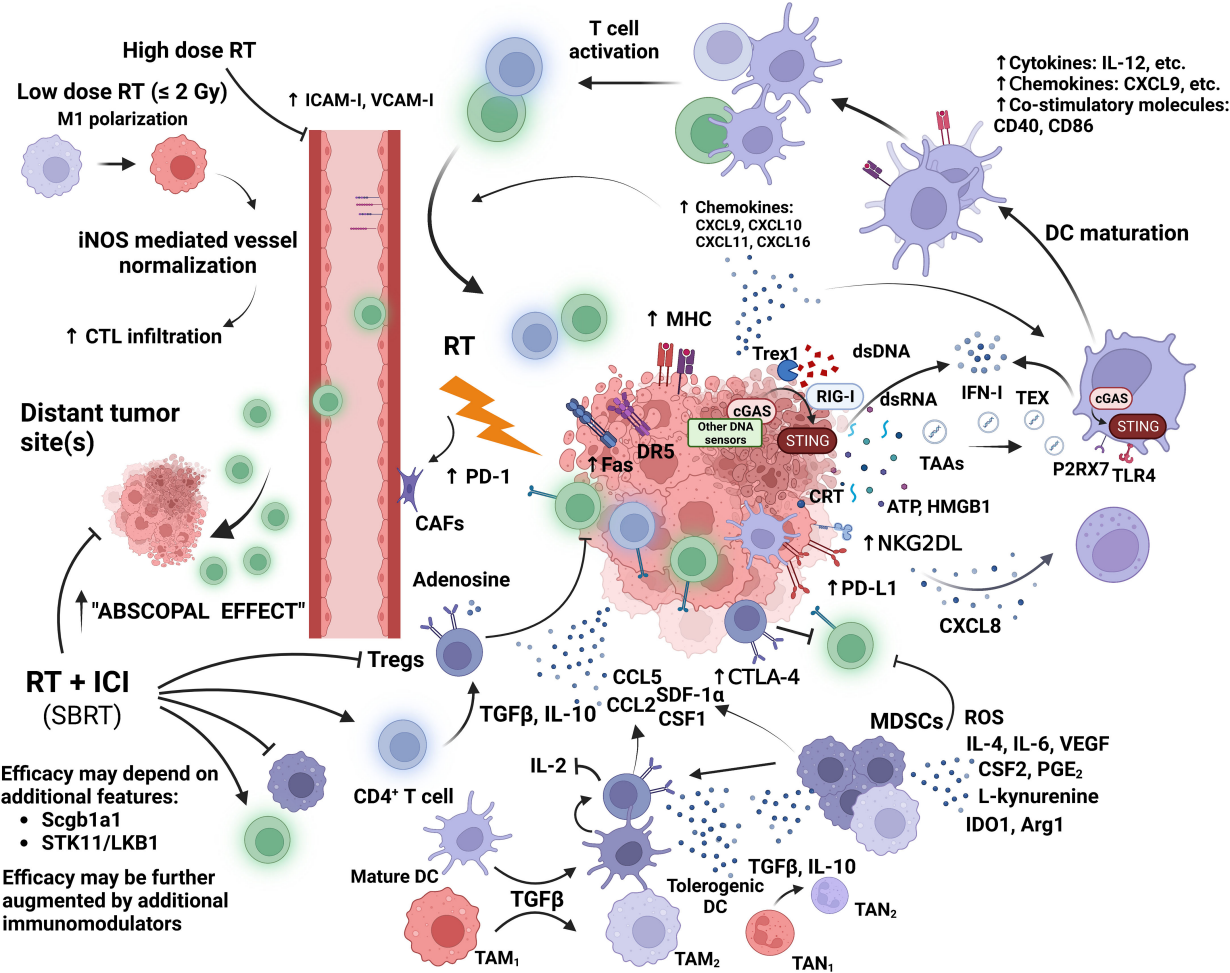
Moderate to high dose irradiation induces the release of tumor associated antigens (TAAs); DNA and RNA fragments; and danger associated molecular patterns (DAMPs) such as calreticulin (CRT), ATP, and HMGB1 by tumor cells. Double-stranded (ds) DNA activates sensors of innate immunity, such as, cyclic GMP-AMP synthase (cGAS); while retinoic acid inducible gene-I (RIG-I) is activated by RNA fragments. These activate the stimulator of IFN genes (STING), which induces IFN-mediated DC recruitment and activation. Cytosolic DNA fragments may be transferred to DCs via tumor derived exomes (TEX). Activated DCs migrate to tumor draining lymph nodes (tdLNs) for cross priming of T cells. Cytosolic dsDNA is degraded by exonuclease Trex1 which is expressed after high dose irradiation of 12-18 Gy. CRT expressed on dying tumor cells leads to DC mediated phagocytosis, while ATP and HMGB1 stimulate DC cross presentation. DCs express a series of cytokines, chemokines, and costimulatory molecules to induce T cell activation. RT upregulates tumor expression of chemokines for T cell homing, and adhesion molecules in the tumor vasculature for T cell extravasation. RT also increases tumor expression of MHC-I and Fas. Fas and DR5 may enhance direct tumor cell killing by CTLs. NK cell cytotoxicity and recruitment are also increased by RT. At low doses, M_1_ polarization is enhanced along with macrophage mediated cytotoxic T lymphocyte (CTL) infiltration due to increased expression of inducible nitric oxide synthase (iNOS) by irradiated, tumor-infiltrating macrophages. These stimulatory effects are offset by the increased tumor-cell release of suppressive cytokines and chemokines, which result in the recruitment of suppressive immune cells and the induction of suppressive immune phenotypes. Leading to the consumption of stimulatory cytokines (e.g., IL-2), further release of suppressive cytokines, and the inhibition of CTLs and NK cells. RT increases the expression of inhibitory checkpoints on effector T cells (T_eff_), DCs, tumor cells, and Tregs within the TIME. High dose RT also induces aberrant tumor vasculature formation, increased fibrosis, hypoxia, and the stimulation of cancer-associated fibroblasts (CAFs). Significant augmentation of CTL infiltration into the local and distant TIME has been observed when RT delivering ablative doses was combined with an immune checkpoint inhibitor (ICI). This was found to be associated with the suppression of Tregs and MDSCs, and the stimulation of CD8^+^ T cell (green) function. The synergy between RT and ICI also depends on specific features of the tumor residing tissue and the tumor itself.

Various mechanisms of immune escape by cancer may lead to poor response to ICIs, which shed light on the development of additional therapeutic strategies to induce an inflamed TIME. As a major local treatment modality for cancer, RT has been shown to have immunogenic properties (21). Ablative doses of radiation can cause immunogenic cell death (ICD) through the stimulation of antigen presentation, leading to increased maturation and recruitment of effector CD8^+^ T cells (**Figure 3**) (108–112). Its immunostimulatory properties may lead to the restoration of an overall inflamed TIME and enhanced antitumor immunity. Furthermore, such immunogenic antitumor effects may be significantly augmented when RT is combined with immunomodulatory agents, such as ICIs (21, 22).

### Immunostimulatory properties of radiotherapy

RT’s ability to induce ICD has been well characterized (21–25). This phenomenon largely depends on its ability to increase the release of tumor-associated antigens (TAAs), double stranded (ds) DNA fragments, and various danger associated molecular patterns (DAMPs) by tumor cells (**Figure 3**). Subsequently, leading to increased antigen presentation, T cell activation and augmented antitumor immunity (22–24). As shown *in vitro*, irradiation increased intracellular protein breakdown and mammalian target of rapamycin (mTOR) mediated peptide production (113). Some of the peptides produced are TAAs generated from radiation-induced immunogenic mutations, which are expressed in a radiation dose dependent manner (114). These TAAs can be CD8^+^ T cell specific, or Th_1_ CD4^+^ T cell specific. Therefore, radiation-upregulated TAAs activate both CD8^+^ and Th_1_ CD4^+^ T cells in a poorly immunogenic tumor, which result in enhanced antitumor response to ICIs and vaccines (114, 115). CD4^+^ neoantigens are essential in radiation-induced antitumor immune response, as they not only result in enhanced CD8^+^ T cell activation and cyto-toxicity, but also engage in direct tumor cell killing through interactions with Fas and the death receptor DR5 on tumor cells (115, 116).

Ionizing radiation (IR) introduces base and sugar damage, crosslinks, and sing or double stranded breaks (SSBs or DSBs) in the DNA (117). The dsDNA breaks represent the most common and lethal radiation induced DNA damage (23). A DNA damage response (DDR) is subsequently elicited by ss or dsDNA breaks, which is mediated by DNA-dependent protein kinase (DNA-PK), ataxia telangiectasia-mutated (ATM), and ataxia telangiectasia and rad-3-related protein (ATR) (24). DNA-PK facilitates non-homologous end-joining (NHEJ), while ATM and ATR facilitate homologous recombination (HR) repair and stabilization of stalled replication forks. Whereas the error-prone NHEJ, and the more precise HR for simple dsDNA breaks are fast, the repairment of more complex DNA damage characterized by multiple oxidative base damage, basic sites, or SSBs around a DSB, is both slower and more error-prone (117).

Defective DNA damage repair leads to the formation of micronuclei from chromosomal fragments through mitosis. The micronuclei are easily ruptured with dsDNA fragments released into the cytosol and detected by the cytosolic dsDNA sensor, cyclic GMP-AMP synthase (cGAS), which localizes to the micronuclei (24). cGAS then activates its adaptor, stimulator of interferon genes (STING), which leads to the secretion of IFN-I and the recruitment of DCs into the TIME (23, 24). Many other sensors of cytosolic nucleic acid exist, such as DDX41, ZBP1, IFI16, MRE11, and HDP-RNP, which can all activate the STING pathway, leading to the initiation of IRF3-dependent or NF-κB-dependent transcriptional programs (118). RNA sensors, such as retinoic acid inducible gene-I (RIG-I) and melanoma differentiation-associated protein 5 (MDA5), can also be activated by radiation, leading to increased IFN-I signaling and DC recruitment (23, 119, 120).

Upon dsDNA sensing within the TIME, intra-tumoral DCs upregulates IFN-β release in a STING dependent manner, which stimulates T cell cross presentation in the tdLNs and T cell activation through the expression of stimulatory cytokines (e.g., IL-6, IL-12, IL-15, TNF), chemokines (e.g., CXCL9), and costimulatory molecules (e.g., CD40, CD86) (121, 122). It is not entirely clear how the cytosolic dsDNA was transported into the cytoplasm of DCs. One mechanism may be through tumor-derived exomes (TEX) (123). The accumulation of cytosolic dsDNA within the tumor cell is regulated by the DNA exonuclease Trex1, which degrades dsDNA. *In vitro*, Trex1 expression is increased at fractional doses between 12-18 Gy, which correlated with significant reduction of cytosolic dsDNA (124).

Irradiation induced cell death and stress also cause the release of many other DAMPs, which are recognized by pattern recognition receptors (PRRs) on APCs, such as Toll-like receptors (TLRs), nucleotide-binding oligomerization domain (NOD)-like receptors (NLRs), and the receptor for advanced glycation end products (RAGE) (125–127). The most commonly identified DAMPs associated with IR induced cell death are cell surface calreticulin, ATP, and the high mobility group box 1 (HMGB1) (127). Calreticulin is a chaperone protein residing in the ER. It translocate to the surface of dying tumor cells to signal to DCs for phagocytosis as an early event in IR-induced ICD (125, 127). As later events during ICD, ATP and HMGB1 are released into the TIME (125). ATP leads to the activation of the NLRP3/ASC/caspase-1 inflammasome by stimulating the purinergic P2RX7 receptors on DCs (128). This leads to the release of IL-1β, which induces CD4^+^ and CD8^+^ T cell cross priming (129, 130). On the contrary, HMGB1 stimulates T cell cross priming through binding to TLR4 on DCs (131).

Other than enhancing antigen presentation, RT also plays a stimulatory role in other key steps of antitumor immunity generation. *In vitro*, the expression of chemokines essential for CD8^+^ T cell recruitment, CXCL9, CXCL10, CXCL11, CXCL16, is significantly elevated after high fractional doses between 10-12 Gy (132–134). Diminished T cell extravasation from aberrant tumor vasculature can be improved by RT due to increased expression of adhesion molecules (108, 135, 136). Increased VCAM-1, and ICAM-1 expression after irradiation with fractional doses of 8-15 Gy have been detected, which resulted in persistent T cell infiltration into the tumor (108, 136). In murine colon and breast cancer mouse models, ICAM-1 expression was significantly elevated after 8 Gy x 3 fractions, which was shown to stimulate a strong CD8^+^ T cell mediated systemic response (136). Multi-fractional(MF) dose regimens with moderate doses may be more suitable for the induction of such effects as they usually lead to more moderate vascular damage that will allow for T cell extravasation. On the contrary, single-fraction (SF) high dose irradiation may lead to significant vessel damage, which prohibits any significant amount of T cell extravasation into the TIME (21, 137).

Normalization of tumor vasculature has been reported at both low or moderate fractional doses (138, 139). Significant T cell recruitment mediated by inducible nitric oxide synthase (iNOS), which is secreted by tumor infiltrating M_1_ macrophages, is observed at doses as low as 2 Gy, due to iNOS regulated T cell transmigration (138). Low dose (LD) irradiation of 1 Gy was further shown to induce a significant increase in the IFN mediated influx of CTLs into a non-inflamed TIME *in vivo*, which represents a potential strategy to induce an inflamed TIME that will lead to increased response to ICIs and other immunomodulators (140). Also, RT can alleviate the impaired tumor cell recognition by CTLs through inducing MHC-I expression on tumor cell surface; and stimulate tumor cell lysis by upregulating Fas expression on tumor cells (113, 141).

RT can stimulate innate anti-immunity mediated by NK cells. Irradiation increased NK cell cytotoxicity and homing in a canine sarcoma model (142). NK cell viability and cytotoxicity are increased with increased fractionation (143). Increased NK cell cytotoxicity post-radiation is due to increased expression of ligands to activating receptor NKG2D on tumor cells that is mediated by STING-dependent dsDNA sensing pathways (144, 145). Secondly, the increased post-radiation NK cell homing is due to increased tumor cell expression and secretion of CXCL8 mediated by NF-κB and mTOR, respectively (146).

### Immunosuppressive properties of radiotherapy

Among all immune cells, CD8^+^ T cells are the most radiosensitive, whereas CD4^+^ T cells and myeloid cells are more radioresistant with macrophages and granulocytes being the most radioresistant (147). This partially accounts for the increased level of suppressive immune cells, such as Tregs, within the TIME post-radiation (148, 149). However, tumor residing CD8^+^ T cells are more radioresistant than naïve and peripheral CD8^+^ T cells, which has attributed to IR-induced upregulation of TGFβ within the TIME (150). This is accompanied by increased CD8^+^ T cell exhaustion, which is evidenced by increased T_eff_ expression of PD-1 and IFN-γ mediated upregulation of tumor PD-L1 expression (151–154).

After irradiation, many cytokines, chemokines and metabolites are upregulated within the TIME, leading to increased recruitment of suppressive immune cells, such as Tregs; which is accompanied by decreased CTL cross priming, recruitment, and function (**Figure 3**) (21–25). As a major mediator of immunosuppression that is being upregulated by irradiation within the TIME, TGFβ is involved in the suppression of CD8^+^ T cell mediated adaptive antitumor immunity in multiple ways (153, 155–157). These mainly involve inhibiting CD8^+^ T cell’s cytolytic function and recruitment; blocking Th_1_ differentiation and inducing a Treg phenotype; directing DC differentiation towards a tolerogenic phenotype; suppressing NK cell function; recruiting monocytes; polarizing macrophages toward an M_2_ phenotype; and mediating anti-CD 8^+^ T cell function by MDSCs (157, 158). Together with IL-10 secreted by T cells and other immune cells within the TIME post-radiation, more CD4^+^ T cells are being converted to Tregs (159–162). *In vivo*, irradiation also increased tumor cell expression of CCL2, which increased Treg recruitment into the TIME (163).

IR-induced increase in Tregs are affected by radiation dose fractionation with higher levels of Tregs observed in the TIME after SF high-dose irradiation of > 10 Gy (164–166). Although MF regimens of lower fractional dose can induce an increase in T cell and NK cell activation early, more latent T cell and NK cell activation was observed with SF ablative doses (166). IR-induced Tregs have higher expression of CTLA-4, which leads to increased inhibition of T cell cross presentation (102, 165, 166). Also, Tregs consume IL-2 within the TIME; release additional suppressive cytokines, including TGFβ, IL-10, IL-35, Fgl_2_; and express ectonucleotidases, such as CD39 and CD73, which increase adenosine production (102).

Increased recruitment of myeloid cells into the TIME post-radiation has been consistently observed. One common mechanism of radiation induced myeloid cell tumor infiltration is increased homing through the upregulation of chemokine expression in tumor cells, such as CCL2, CCL5, and HIF-1 induced stromal-derived factor 1α (SDF-1α), which interacts with CCR2, CCR5, and CXCR4 (163, 167–170). Increased CCL2-CCR2 mediated chemotaxis is found to be at least partially induced by STING mediated IFN-β signaling (171). Irradiation also stimulates DNA damage-induced kinase ABL1 mediated upregulation of macrophage colony-stimulating factor (M-CSF or CSF1) in tumor cells (172). This subsequently increases CSF1-CSF1R mediated trafficking of myeloid cells into the TIME (172, 173).

Suppressive myeloid cells originate from myeloid progenitor cells in the bone marrow, which migrate to peripheral organs to develop into macrophages, DCs, or granulocytes (174). However, soluble factors produced within the TME promote local accumulation and activation of MDSCs. While MDSCs play a suppressive role within the TIME, functional differentiation TAMs and TANs depends on the balance of immunostimulatory and immunosuppressive signals within the TIME (175). Upon irradiation, increased suppressive myeloid cell infiltration generally impairs CTL mediated anti-tumor immunity through nutrient depletion, increasing oxidative stress, impairing CTL trafficking and function, and stimulating Treg recruitment and function (174, 175).

MDSCs within the TIME are primarily categorized as granulocytic (g)/polymorphonuclear (PMN)-MDSCs or monocytic (M)-MDSCs (176). MDSCs secrete a series of suppressive solutes, such as IL4, IL10, TGFβ1, CSF2, VEGFA, PGE_2_ and L-kynurenine. VEGFA, TGFβ1, IL-10, and IL-6 upregulate Treg expansion and TAM M_2_ polarization. MDSCs overexpress IDO1 and Arg1, leading to depletion of key amino acids, such as arginine, cystine, and tryptophan within the TIME. MDSCs also produce reactive oxygen species (ROS) and reactive nitrogen species (e.g., nitric oxide, NO), leading to modified MHC-I and receptors for antigens and chemokines on T cells (175, 176). PD-L1 expressed on MDSCs directly suppresses CTLs and NK cells (176).

In addition to myeloid cell trafficking, CSF1R signaling also mediates TAM polarization toward an M_2_ phenotype (177). High dose (HD) irradiation induces the expression of M_2_ associated genes, such as Arg1, and COX2, whereas LD irradiation of 0.5-2 Gy was shown to induce M1 polarization in TAMs (138, 140, 178). M_2_ TAMs induce immunosuppression in ways similar to MDSCs. On the contrary, M_1_ TAMs produce proinflammatory cytokines, such as TNF and IL-12; recruit Th_1_ CD4^+^ T cells through CXCL9 and CXCL10 secretion; and induce direct tumor cell killing (175). Similar to TAMs, TANs polarize into the antitumoral N_1_ or the protumoral N_2_ phenotypes with N_2_ TANs having functional overlap with PMH-MDSCs (179, 180). TANs within the TIME have generally been associated with an immunosuppressive role and poor response to immunotherapy or RT with GLUT1 identified to be essential to their protumoral role (180–183).

Increased release of suppressive cytokines post-radiation, such as VEGF and TGFβ, may further stimulate the development of an immunosuppressive TIME by inducing aberrant tumor vasculature formation, increasing fibrosis, and activating CAFs, resulting in poor perfusion, increased hypoxia, increased recruitment of suppressive immune cells; and the inhibition of CTL function, homing, and endothelial adhesion (87, 184–187). However, how RT influences CAFs’ immunomodulatory function remains controversial and needs to be better defined (188).

## Augmenting antitumor immunity by combining radiotherapy with immunomodulatory agents

Immunostimulatory properties of radiation allows it to act synergistically with immunomodulatory agents to induce a CD8^+^ T cell mediated immunogenic antitumor response that is stronger than from either alone (**Figure 3**). Strong local and systemic antitumor response (response outside of the irradiated field, which is also known as the “abscopal effect”) were elicited when RT is combined with immunomodulatory agents stimulating the proliferation and/or activation of DCs, Th_1_ CD4^+^ T cells, and CD8^+^ T cells (189–192). This was due to IFN-I mediated DC infiltration, CD8^+^ cross priming, and subsequent effector CD8^+^ T cell infiltration into the TIME (191, 192). Such antitumor response was consistently observed when RT was combined with an anti-CTLA-4 ICI *in vivo* (193–196). The intensity of an abscopal response generated from such combinations are dose fractionation and treatment sequence dependent. As shown by Dewan et al, an abscopal effect was most prominent after combining an anti-CTLA-4 antibody with 8 Gy x 3 fractions vs. 6 Gy x 5 fractions, or 20 Gy x 1 fraction with SF irradiation failing to induce any such effect (194). The three-fraction regimen was further shown to have stronger distant effect than the five-fraction regimen. When compared with concurrent delivery of RT and an anti-CTLA-4 ICI, ICI delivery after RT was shown to have reduced distant therapeutic effect. Increased antitumor immunity with the combined treatment is associated with increased CTLs, CD8/CD4 ratio, reduced Tregs within the TIME, and increased CD8^+^ T cell clonality (195, 196).

Unlike anti-CTLA-4 ICIs, which suppress the inhibition of CD8^+^ T cell activation within the more proximal site of tdLNs, anti-PD-(L)1 ICIs are more recognized for their ability to overcome PD-(L)1 mediated inhibition of CTL function within the peripheral TIME (6). PD-L1 expression is upregulated in DCs and tumor cells after irradiation, while acute decrease in PD-1 expression in CD8^+^ T cells within the TIME is observed after RT with an ablative dose (197, 198). Significant improvement in local and abscopal antitumor responses was observed after combined treatment with RT and an anti-PD-L1 ICI (197–199). Increases in response were more prominent with hypofractionated schedule delivering ablative doses, which results in increased CD8^+^ T cell infiltration & function; and reduced intratumoral MDSCs resulting from decreased trafficking and CD8^+^ T cell mediated direct killing requiring TNFα (197, 199). After ablative doses, latent increase in PD-1 expression by tumor-infiltrating T cells and elevated PD-L1 expression by tumor cells at both primary and secondary tumor sites were observed (200, 201). This was accompanied by decreased intratumoral Tregs.

Anti-PD-1 ICIs combined with RT induced significant increases in tumor-antigen specific and memory CD8^+^ T cells, as well as CD8^+^/Treg ratio within the peripheral TIME, subsequently leading to significantly amplified local and abscopal antitumor responses (201–204). The increased CD8^+^/Treg ratio mainly resulted from a reduction of Tregs within the peripheral TIME after the combined treatment, which is accompanied by increased CD8^+^ T cell clonality at both primary and secondary tumor sites (202, 203). Contrary to the reduction of intratumoral MDSCs observed after ablative doses, increased intratumoral accumulation of MDSCs has been observed after combined treatment with conventionally fractionated radiation and an anti-PD-1 antibody (203). Overall, pre-clinical data supports the use of hypofractionated RT schedules in combined RT and anti-PD-(L)1 treatment strategies.

The benefit of RT and anti-PD-(L)1 combinations also depends on features of the tumor residing tissue and the tumor itself. For example, LD irradiation with 4 Gy x 3 fractions may induce significant CD8^+^ T cell tumor infiltration and increased CD8^+^ T cell function when combined with an anti-PD-1 antibody (205). This combination led to significant antitumor response in a KRAS^G12D^ mutant orthotopic mouse model of lung adenocarcinoma, which depended on the presence of lung tissue residing club cells that express synaptosome-associated protein 23 (Scgb1a1). This is due to club cell secretory proteins’ immunostimulatory roles, such as suppression of myeloid cells (205). Alternatively, STK11/LKB1 mutations, which frequently co-occurs with KRAS-mutations in lung adenocarcinomas, were associated with poor synergy between RT and anti-PD-1 *in vivo* (200). This may result from increased CTL exhaustion in the presence of increased CD8^+^ T cell infiltration and CD8^+^/Treg ratio after the combined treatment. These examples demonstrate the influences of various tumor-related features on the TIME, which may dictate tumor response to combined RT and ICI treatment. Additional therapeutic strategies are needed to further augment the efficacy of RT and ICI combinations in these situations.

The PD-1/PD-L1 axis is a main mediator of CD8+ T cell exhaustion with in the TIME of resistant melanomas after combined treatment with RT and an anti-CTLA-4 ICI (206). In resistant tumors, increased tumor expression of PD-L1 and the proportion of PD-1^+^ EOMES^+^ CD8^+^ T cells that do not express markers for activation, Ki67 and GzmB were found. Activated CD8^+^ T cells and CD8^+^/Treg ratio markedly increased within the TIME after an anti-PD-L1 ICI was added to the RT and anti-CTLA-4 ICI combination. Dual CTLA-4 and PD-(L)1 blockade combined with radiation led to a CR rate of 80% and significant prolongation of survival *in vivo*. Therefore, significant antitumor immunity may be generated by combining RT and the targeting of multiple stimulatory and/or inhibitory checkpoints. This strategy may be further explored in the context of combining RT with dual immune checkpoint blockade (ICB) (207–212).

Further improvement of antitumor response was observed when an OX40 agonist, an anti-TIM-3, or an anti-TIGIT antibody was added to the RT and anti-PD-(L)1 antibody combination (210–212). Significant increase in CD8^+^ T cell and CD103^+^ DC infiltration, as well as decreased CD8+ T cell exhaustion were observed with the addition of an OX40 agonist (210). This led to further improvement in local and abscopal responses, leading to an survival advantage over radiation combined with an anti-PD-1 antibody alone. Similar survival advantage was observed with the addition of an anti-TIM-3, or an anti-TIGIT antibody (211, 212). The added benefit from an anti-TIGIT antibody was dependent on radiation dose fractionation. TIGIT expression by CD8^+^ T cells was increased by 8 Gy x 3 fractions, but decreased by 2 Gy x 18 fractions (212). Other strategies to further enhance immunostimulatory effects of RT combined with ICIs may include addition of other immunostimulatory agents, such as stimulatory cytokines (e.g., IL-2, IL-12) and activators of innate immunity sensor (213–218). Alternatively, additional therapeutic advantage may be gained by adding agents reducing the level of immunosuppression within the TIME to RT and ICI combinations, such as antibodies against TGFβ, VEGF, or PI3K; M_2_ TAM or MDSC reducing agents; inhibitors of suppressive metabolite production, such as CD73 antibodies; and inhibitors to other checkpoints, such as CD47 antibodies (199, 209, 219–224).

## Clinical overview on combining RT and ICI(s) with a focus on NSCLC

### Induction of an abscopal response with SBRT + ICI(s)

In case report format, dramatic abscopal response (AR) was initially reported in patients with metastatic melanoma or NSCLC who progressed after multiple courses of systemic therapy (225–227). These patients received single-site hypofractionated RT, or stereotactic body radiotherapy (SBRT) either after progression while on an ICI, or concurrently with an ICI after progression on chemotherapy. Abscopal response rates (ARRs) of 51-53% were further identified in small cohorts of patients with advanced melanoma who progressed an anti-CTLA-4 ICI (Ipilimumab) or an anti-PD-1 ICI (Nivolumab or Pembrolizumab) after they received intra- or extra-cranial hypofractionated RT (228, 229). Furthermore, an AR was associated with improved median survival in patients who progressed on Ipilimumab (22.4 vs. 8.3 months, p = 0.002) (228). This association was also corroborated in a prospective trial testing the efficacy of combining hypofractionated RT with a DC stimulating agent in patients with metastases that progressed on conventional systemic therapy (230). In this study, an ARR of 26.8% was observed in 41patients (4 NSCLC, 5 breast cancer, 2 thymic cancer).

Similar ARR’s were observed in early phase trials evaluating the efficacy and safety of administering SBRT sequentially or concurrently with ICIs in patients with metastatic melanoma, or mixed histology solid tumors refractory to conventional systemic therapy (**Table 1**) (231–233). Such systemic response was correlated with the expression of IFN-γ associated genes (232). Among responders to such combinations, CD8^+^ T cells and their expression of 4-1BB & PD-1 were increased in the peripheral blood, reflecting an increase in systemic antitumor immunity after SBRT (233). Also, the intensity of antitumor immunity generated by SBRT combined with an ICI was affected by the choice of site to be irradiated, with liver correlating to higher levels of T cell activation than the lungs.

**Table 1:** Abscopal response following RT + ICIs in metastatic solid tumors.

		Study Type	N	Prior Rx	ICI		RT Dose	ICI Sequence	#Sites Treated	Abscopal Response (AR)	Other Response
Melanoma											
Grimaldi et al. (228)		Retrospective	21	Y	Ipi		20-24 Gy x 1 Frx	Upfront till progression	Mostly 1 site	PR 43% + SD 10%	AR is associated with local response (11/13)
							3-4 Gy x 5-10 Frx 2 Gy x 25 Frx			47.8% in soft tissue & lymphoid sites	mOS (AR *vs.* no AR): 22.4 vs. 8.3 months, *p* = 0.002
Roger et al. (229)		Retrospective	Early Group: 15	Y	Nivo or Pembro		Median: 26 Gy/ 3-5 Frx	Con	Multiple	CR: 20%; PR: 16%; SD: 12%	mPFS (early vs. late RT): 3.0 vs. 16.2 months
			Late Group: 10					Upfront till progression		Early vs. Late RT:	
										CR + PR: 34% vs. 40%	
										SD: 0% vs. 30%	
Sundahl et al. (231)		Phase I	13	Y	Ipi		8-12 Gy x 3 Frx	Neoadj, Con, Adj	1	23% (3/13)	LF: 8% (1/12)
											mOS: 74 weeks
Mixed Histology											
Luke et al. (232)		Phase I	68/73	Y	Pembro		10-15 Gy x 3 Frx	Adj	Median: 2 (2-4)	26.90%	mPFS: 3.1 months
							10 Gy x 5 Frx				mOS: 9.6 months
								
Tang et al. (233)		Phase I	35	Y	Ipi		12.5 Gy x 4 Frx	Con; Neoadj, Con	1	PR + SD: 23%	mPFS: 3.2 months
							6 Gy x 10 Frx				mOS: 10.2 months
NSCLC											
Formenti et al. (234)		Pilot	39 (*21)	Y	Ipi		9.5 Gy x 3 Frx	Con	1	ITT ORR: 25.6% (10/39)	ORR: 18% (33%*);
							6 Gy x 5 Frx			ORR: 50% (10/20)	Rx completion & Disease control improved mOS
											(13 & 20.4 vs. 3.0 & 3.5 months, p < 0.05)
Bauml et al. (235)		Phase II	45	N	Pembro		Unknown	Adj	All sites	Not reported	mPFS: 19.1 months
									(≤ 4 oligomets.)		mOS: 41.6 months.
Qin et al. (236)		Pilot	12	Y	Atezo		8 Gy x 3 Frx	Neoadj, Con, Adj	1	Not reported	3/12 PR; 3/12 SD
							6 Gy x 5 Frx				
Theelen et al. (237)		Phase II	78 (*76)	Y (ICI naïve)	Pembro		8 Gy x 3 Frx	Adj	1	Not reported	ITT ORR_12wks_: 36% vs. 18%, *p* = 0.07
											DCR_12wks_: 64% vs. 40%, *p* = 0.04
											SBRT enhanced PFS & OS in PD-L1 (-) subgroup HR 0.49 & 0.48, p < 0.05
Welsh et al. (238)		Phase I-II	100 (*79)	Both	Pembro		12.5 Gy x 4 Frx	Con, Adj	1-4	ORR(_SBRT vs. Trad. RT)_: 38% vs. 10%	No change in PFS vs. Pembrolizumab alone.
							3 Gy x 15 Frx			ORR(_Salv SBRT vs. Trad. RT_): 33% vs. 17%	
Chen et al. (239)		Retrospective	17/16^a^	Both	Ipi		12.5 Gy x 4 Frx	Ipi: Neoadj, Adj	1-4	Not reported	PFS (Ipi vs. Pembro) HR 3.126, *p* = 0.02
					Pembro		6 Gy x 5 Frx	Pembro: Con, Adj			OS (Ipi vs. Pembro) HR 2.401, *p* = 0.08
Theelen et al. (240)		Pooled Phase II	Pembro + SBRT: 72	Both	Pembro		8-12.5 Gy x 3-4 Frx	Con, Adj; Adj	1-4	ARR: 41.7% vs. 19.7%, *p* = 0.0039	mPFS: 9.0 vs. 4.4 months, *p* = 0.045
		SBRT + P vs. P	Pembro: 76				3 Gy x 15 Frx			ACR: 65.3% vs. 43.4%, *p* = 0.0071	mOS: 19.2 vs. 8.7 months, *p* = 0.0004
Bestvina et al. (241)		Phase I	18/19^b^	N	Ipi + Nivo		10-15 Gy x 3-5 Frx	Con, Adj or Adj	1-4	ORR: 33.3%; DCR: 70.8%	ORR (Con vs. Seq): 44.4% vs. 47.4%
											DCR (Con vs. Seq): 72.2% vs. 52.6%

N, patient number; Rx, treatment; ICI, immune checkpoint inhibitor; RT, radiotherapy; Y, yes; Ipi, Ipilimumab; Gy, Gray; Frx, fraction; PR, partial response; SD, stable disease; mOS, median overall survival; Nivo, Nivolumab; Pembro, Pembrolizumab; Con, concurrent; CR, complete response; mPFS, median progression-free survival; Neoadj, neoadjuvant; Adj, adjuvant; LF, local failure; *, number of patients evaluated; ITT, intent to treat; ORR, objective response rate; Oligomets, Oligometastases; Atezo, Atezolizumab; wks, weeks; DCR, disease control rate; HR, hazard ratio; Trad., traditional; a, #Ipi/#Pembro; ARR, abscopal response rate; ACR, abscopal control rate; b, concurrent/sequential; N, no; Seq, sequential.

SBRT and ICI combination’s ability to induce systemic antitumor immunity in mostly treatment refractory metastatic NSCLC has been intensely investigated in early phase clinical trials (234–241). Despite limited sample size, responses at both irradiated and non-irradiated tumor sites were consistently observed in these studies (**Table 1**). After combing an ICI with RT to mostly 1-2 tumor sites, an ARR between 33-50% were observed (234, 238, 240). Combining dual CTLA-4 and PD-1 blockade with SBRT does not seem to further increase the ARR (241). Clinically, the generation of a robust systemic antitumor response is also associated with the expansion of tumor neoantigen specific CD8^+^ T cells (234).

Administration of Pembrolizumab after local ablative therapy to all oligometastatic sites has led to impressive median progression-free survival (PFS) and overall survival (OS) of 19.1 and 41.6 months, respectively (235). Although a survival advantage was not demonstrated when SBRT delivering 8 Gy x 3 fractions to 1 tumor site was combined with adjuvant Pembrolizumab compared with Pembrolizumab alone in a phase II trial (PEMBRO-RT), noticeable improvement of the objective response rate (ORR) and disease control rate (DCR) at 12 weeks was observed (237). In the subgroup analysis, significant improvements in the PFS and OS were observed in the PD-L1 negative patients, which will be further confirmed in a separate phase II/III study (242). As shown in a phase I/II study, SBRT induced higher ARR (concurrent: 38% vs. 10%; sequential: 33% vs. 17%) than more protracted course of RT when combined with PD-1 blockade (238). In a small cohort, PD-1 blockade was also found to be more suitable than CTLA-4 blockade for combining with SBRT, as it led to significant improvements in PFS and OS (239). The failure to demonstrate a survival advantage in the PEMBRO-RT trial may stem from the suboptimal sample size of the study (N = 78) (237).

After pooling the PEMBRO-RT trial with another similarly designed phase II trial, significant improvements in the ARR and abscopal control rate (ACR) at 12 weeks were observed when SBRT was combined with Pembrolizumab vs. Pembrolizumab alone, which translated into significant improvements in PFS and OS (**Table 1**) (240). While early clinical experience does not demonstrate any significant difference in clinical response rates between concurrent or adjuvant ICI administration with SBRT, concurrent treatment was associated with significantly improved global antitumor response, and survival in patients with non-inflamed NSCLC in the presence of high aneuploidy and low TMB (241, 243). Therefore, valid biomarkers for a noninflamed TIME may be used to effectively select patients for concurrent SBRT and ICI.

### Combining an ICI with chemoradiation in earlier stage NSCLC

Combining an ICI with chemoradiation for stage II-III NSCLC has led to significant reduction in distant metastasis or death, leading to prolonged PFS and OS (**Table 2**) (244–250). Adjuvant Durvalumab after concurrent chemoradiation delivering mostly 60-66 Gy in 30-33 fractions in stage III NSCLC led to remarkable improvement in median PFS from 5.6 to 16.9 months, and median OS from 29.1 to 47.5 months, respectively (244, 251, 252). This was associated with increased ORR (29.8% vs. 18.3%) and a 41% reduction in the risk of death and distant metastasis at 5 years (244). Similar findings were reported when other anti-PD-(L)1 agents were administered after chemoradiation (245–247). As shown in a subgroup analysis of the PACIFIC trial, such OS advantage may be limited to patients with PD-L1 expression ≥ 1% (252). A survival benefit of lesser scale was also observed if patients received sequential chemoradiation prior to adjuvant anti-PD-L1 ICI. ORR in patients receiving sequential chemoradiation prior to ICI administration appears less than that observed in patients who received concurrent chemoradiation (247). This may be one reason for the relatively less survival benefit with adjuvant anti-PD-L1 ICI observed in GEMSTONE-301, in which 33-34% of patients received sequential chemoradiation prior to Sugemalimab (245).

**Table 2:** Combining chemoradiation with ICI in early-stage NSCLC.

Study	Treatment Schema	N^a^	Stage	Clinical Outcome
Phase III				
PACIFIC Trial (244)	cCRT + Adj Placebo	236/237	III	ORR: 29.8% vs. 18.3%, *p* < 0.05
	cCRT + Adj D x 1 yr	473/476		TTDM: HR 0.59, 95% CI 0.47 - 0.74, *p* < 0.05
				Incidence of new lesions: 24.2% vs. 33.3%, *p* < 0.05
				PFS: median 16.9 vs. 5.6 months, 5yr 33.1% vs. 19.0%, *p* < 0.05
				OS: median 47.5 vs. 29.1 months, 5yr 42.9% vs. 33.4%, *p* < 0.05
GEMSTONE-301 (245)	Con/Seq CRT + Adj Placebo	126 (85/41)	III	PFS: median 9.0 vs. 5.8 months, *p* = 0.0026; 1yr 45.4% vs. 25.6%
	Con/Seq CRT + Adj S x 2 yrs	255 (169/86)		OS: median NR vs. 24.1 months, *p* = 0.0009
Phase II				
LUN 14-179 (246)	Con CRT + Adj P x 1 yr	92/93	III	TMDD: median 30.7 months; 3yr 49.9%
				PFS: median 18.7 months; 3yr 37.4%
				OS: median 35.8 months; 3yr 48.5%
PACIFIC-6 (247)	Seq CRT + Adj D x 2 yr	117	III (1 IA)	ORR: 17.1%; median DoR: NR
				PFS: median 10.9 months; 1yr 49.6%; OS: 2y 69.8%
KEYNOTE-799 (248)	Ind Chemo-P x 1 cycle + cCRT-P	214/216	III	A: ORR: 70.5%, DCR: 88.4%; PFS: median NR; OS: median NR
	Adj P x 1 year	A 112; B 102		B: ORR: 70.6%, DCR: 93.1%; PFS: median NR; OS: median NR
ETOP NICOLAS (249)	Ind Chemo x 1 cycle + cCRT-N	74/79	III	ORR: 73.4%, CR: 6.3%; DoR: 11.0 months; TTF: median 9.2 months
	Adj N x 1 yr			PFS: median 12.7 months, 1yr 53.7%; OS: median 38.8 months, 2yr 63.7%
DETERRED (250)	cCRT-A + Adj A x 1 yr	30/37	IIB-IIIC	PFS: median 13.2 months; OS: median NR

a, treated/randomized; Con, concurrent; CRT, chemoradiation; Adj, adjuvant; D, Durvalumab; yr, year; ORR, objective response rate; TTDM, time to death or distant metastasis; HR, hazard ratio; CI, confidence interval; PFS, progression-free survival; OS, overall survival; Seq, sequential; S, Sugemalimab; NR, not reached; P, Pembrolizumab; TMDD, time to metastatic disease or death; Ind, induction; cCRT, concurrent chemoradiation; A, squamous/nonsquamous; B, squamous; DoR, duration of response; N, Nivolumab; TTF, time to failure; A, atezolizumab.

Concurrent administration of an anti-PD-(L)1 agent with chemoradiation has been shown to be tolerable (253). High ORR of approximately 70% was observed with concurrent administration of chemoradiation and an anti-PD-1 agent (248, 249). However, concurrent administration appears to be associated with higher incidences of severe toxicity and/or fatal events when compared with those observed in the PACIFIC trial, which was associated with ≥ grade 3 adverse effects of < 10%, and no fatal toxicity (248–250, 254). This may narrow the therapeutic window associated with the concurrent strategy. How it compares to sequential administration of concurrent chemoradiation and adjuvant ICI is being further tested in the phase III randomized trial, ECOG-ACRIN EA5181 (255). Neoadjuvant administration of moderate dosed SBRT to the primary tumor combined with Durvalumab has also been shown to significantly increase the major pathological response (MPR) rate comparing to Durvalumab alone (53.3% vs. 6.7%, crude odds ratio, OR: 16, *p* < 0.0001) (256). MPR in the combination group led to an impressive CR rate of 50%. The role of combining SBRT and immunotherapy in very early staged NSCLC is currently being investigated in multiple ongoing trials (257–260).

### Combining RT with an ICI in other solid tumors

Combining RT with immunotherapy and chemotherapy has also been tested in small cell lung cancer (SCLC). This strategy was found to be feasible in metastatic or localized stages (261, 262). In limited stage SCLC, concurrent chemoradiation and Pembrolizumab was found to be tolerable, and led to a median OS of 39.5 months, which compares favorably with the median survival of 28.5 months after the same chemoradiation regimen alone in RTOG 0538 (262, 263). In a trial designed similarly to the PACIFIC trial, adjuvant Nivolumab in stage II-III esophageal or gastro-esophageal junction cancer patients with pathological residual disease after neoadjuvant chemoradiation led to improved median disease-free survival (DFS) from 11.0 to 22.4 months (264). On the contrary, combining either SBRT or chemoradiation with an anti-PD-(L)1 antibody has not been shown to be advantageous in head and neck cancer (265, 266). The underlying mechanism for this lack of synergy remains unclear.

## Novel treatment concepts in combining radiotherapy and immunotherapy

### Incorporation of LD-RT in RT and ICI combinations

LD-RT may induce tumor vasculature normalization and TAM M_1_ polarization, leading to increased tumor infiltration by CTLs (138, 140). Significant increase in Th_1_ CD4^+^ mediated antitumor immunity has been observed when LD-RT is combined with immunotherapy ± chemotherapy (140, 267). Response to such combinations in humans was associated with increased TCR clonality in the peripheral blood, which implies the upregulation of systemic antitumor immunity (140). However, combining LD-RT or moderate doses of 8 Gy x 3 fractions with CTLA-4 and PD-L1 dual blockade did not lead to added clinical benefit in poorly immunogenic colorectal cancer (CRC) and NSCLC in phase II clinical trials (268, 269). This may be due to suboptimal intensity of the antitumor immunity generated by LD-RT to a single site alone, and the induction of suppressive immune cells (270). Multi-site irradiation may provide a solution to this problem.

*In vivo*, LD-RT with targeted radionuclide therapy (TRT) that targets all malignant lesions was able to induce strong local and abscopal effects when combined with an anti-CTLA-4 ICI, leading to improved survival than either treatment alone (271). In addition to stimulatory effects on NK cells and myeloid cells, CD8^+^/Treg ratio increased more 1 day after TRT than after either focal LD-RT or moderate dose RT. Combining TRT and ICI led to significant increase in T_eff_ infiltration, CTL activation, and reduction of CD8^+^ T cell exhaustion & IL-10 within the TIME of poorly immunogenic tumors. This phenomenon was shown to be STING-dependent (271). When combined with focal high-dose (HD) RT and ICI, TRT led to the best response at the primary and secondary sites, which further improved survival compared with either HD-RT or TRT alone combined with ICI. Thus, LD-RT and HD-RT’s different immunostimulatory effects can supplement each other. Comparing to HD-RT alone, further decreases in Tregs, IL-10 secreting macrophages, and TGFβ within the TIME of secondary sites were observed when LD-RT to metastatic sites was added (272, 273).

Combining LD-RT to secondary tumor sites with focal moderate-to-high dose RT to the primary tumor further augmented local and abscopal responses when they are combined with CTLA-4 and PD-1 dual blockade *in vivo* (273). Such effects were NK and CD4^+^ T cell dependent, and significantly improved survival. Similarly, enhanced systemic antitumor immunity was observed when LD-RT to secondary sites was combined with moderate-dosed RT and an anti-PD-1 ICI (274). The triple combination increased CD8^+^ T cell recruitment and reduced intratumoral MDSCs. The benefit of such triple combination was confirmed in small patient cohorts, in which LD-RT to distant lesions led to significant response in irradiated distant lesions (273–275). In a phase II clinical trial, improved response in metastases from NSCLC and melanoma was observed after such treatment in patients who progressed on an anti-PD-(L)1 or anti-CTLA-4 ICI, or their combination (276). Thus, combining LD-RT, HD-RT, and ICI represents a potential strategy to induce an inflamed TIME at both primary and distant tumor sites that are “cold”.

### Combining RT with adoptive cell therapy

The immunostimulatory effects of hypofractionated RT may be augmented by altering the immune cell composition of the TIME. This may be achieved by directly targeting suppressive immune cells, such as Tregs; or administering stimulatory immune cells, such as Th_1_ CD4^+^ T cells (277–279). Adoptive T cell therapy (ACT), which includes chimeric antigen receptor (CAR)-T cell therapy or the administration of engineered-TCR cells, may be further enhanced by RT. This is due to RT’s ability to induce TAA release, IFN-I mediated antigen presentation, and CD8^+^ recruitment at high doses, as well as its ability to normalize tumor vasculature, alter local stroma, and polarize TAMs toward the M_1_ phenotype without excessive cytotoxic effect on intratumoral CTLs at low doses (138, 280, 281).

Hypofractionated RT stimulates the proliferation and activation of both tumor antigen specific and adoptively transferred T cells, homing of CD8^+^ T cells, and tumor cell susceptibility to direct T cell cytotoxicity (134, 282). These stimulatory effects led to improved survival *in vivo* (134). A major limitation to ACT’s efficacy, T cell recruitment into the TIME, may be improved by RT due to its ability to stimulate CCL5, CXCL9, CXCL10 expression that is at least partially mediated by RT induced recruitment of eosinophils (282). As shown in 3 cohorts of NSCLC and nasopharyngeal cancer patients, RT significantly increased peripheral eosinophil concentration, which correlated with significantly improved PFS.

CAR-T cell therapy’s efficacy relies on tumor expression of specific antigens, which may be lost through immune editing (90). This challenge may be overcome by LD-RT priming, which was shown to induce TRAIL-mediated tumor cell killing in the absence of the CAR specific antigen with excellent local response (283). This makes LD-RT priming a potential therapeutic strategy to overcome CAR-T cell therapy resistance. When used as a debulking regimen in relapsed non-Hodgkin’s lymphoma (NHL), regional RT was shown to be correlated with dramatic response to CAR-T cell therapy and much less toxicity than chemotherapy in a small cohort of 10 patients (284). Synergistic effects between NKG2D-based CAR-T cell therapy and RT were also observed in a glioblastoma mouse model (285). RT may augment ACT’s therapeutic efficacy at either low and high doses, and how to take advantage of their unique immunostimulatory properties remains to be further investigated (286).

## Conclusion

The TIME is sculpted by the tumor’s intrinsic features and those of the various immune cells, stromal cells, endothelial cells within the TME. Various mechanisms of failure to generate anti-tumor immunity exist, resulting in a noninflamed or “cold” TIME which is associated with primary or acquired resistance to ICIs and other immunomodulatory agents. Radiotherapy’s immunostimulatory effects are offset by its immunosuppressive effects. Its immunostimulatory effects may be augmented when combined with ICIs and other immunomodulators in a manner that is both dose/fractionation and sequence dependent. This strategy may stimulate the generation of antitumor immunity, and/or reduce immunosuppression caused by suppressive mediators and cells within the TIME. Therefore, combining RT with immunotherapy may help overcome resistance to ICIs and other forms immunotherapy, and enhance established treatments for cancer with promising early clinical evidence emerging. In addition, strategies to enhance the efficacy of such combinations warrant further exploration.

## Author contributions

AC and NN contributed to conception and design of the study. AC wrote the first draft of the manuscript. All authors contributed to the article and approved the submitted version.
